# Supplementary figures and images for: Long-term in vitro monitoring of AAV-transduction efficiencies in real-time with Hoechst 33342
Source: PLoS One. 2024 Mar 1;19(3):e0298173. doi: 10.1371/journal.pone.0298173 (PMC10906819; doi:10.1371/journal.pone.0298173)

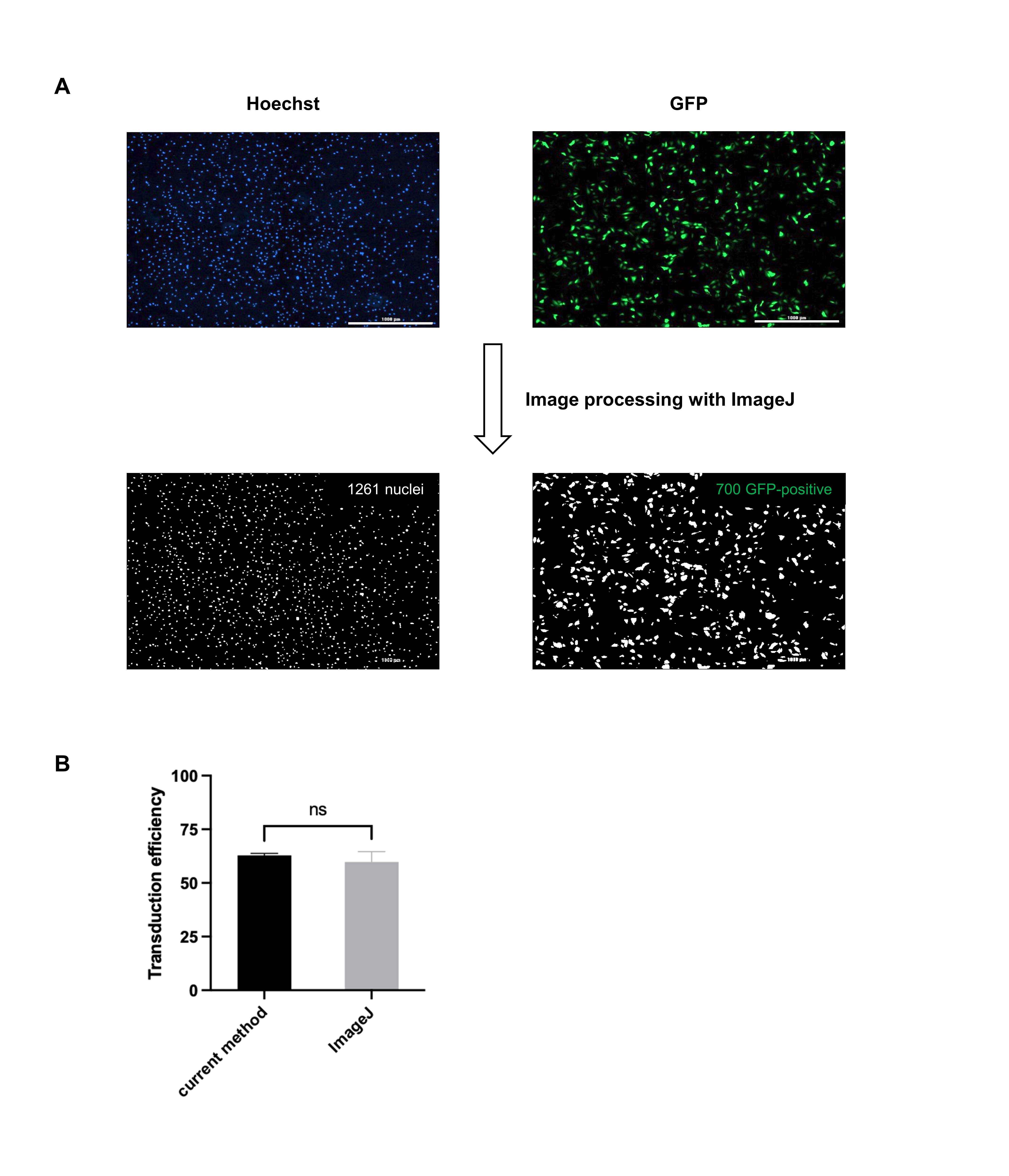

Supplement: S1 Fig — The fluorescent images were analyzed with ImageJ. (A) Representative raw and analyzed images of Hoechst and GFP channels. The scale bars of 1000 μm were represented. (B) Transduction efficiencies of three technical replicates were calculated with ImageJ and our proposed method. Ns, non-significant. (TIF) [file pone.0298173.s002.tif]

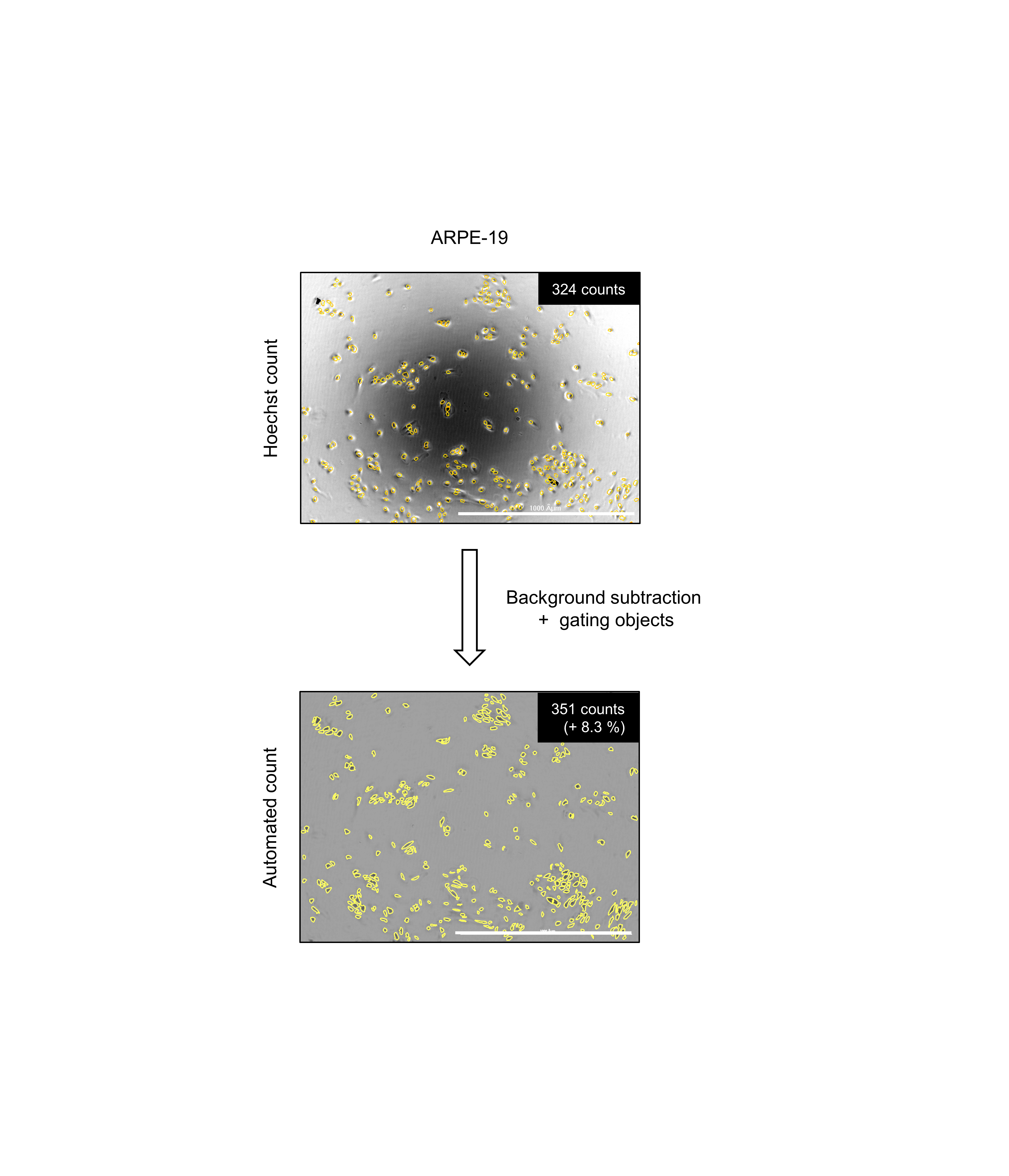

Supplement: S2 Fig — The top picture shows PC image of the Hoechst-gated nuclei (marked in yellow). The bottom picture shows the PC image after background subtraction with automated gated objects (highlighted in yellow). The scale bar represents 1000 μm. (TIF) [file pone.0298173.s003.tif]
