# Supplementary material for: Long-term in vitro monitoring of AAV-transduction efficiencies in real-time with Hoechst 33342
Source: PLoS One. 2024 Mar 1;19(3):e0298173. doi: 10.1371/journal.pone.0298173 (PMC10906819; doi:10.1371/journal.pone.0298173)
Supplement: Attachment — Submitted filename: Response to Reviewers.docx [file pone.0298173.s005.docx]

**Forschungslabor Experimentelle Augenheilkunde**

**Dr. rer. nat Heiko Fuchs, Laborleiter**

OE6120

Heiko Fuchs

Telefon:	0511 532-7246

Fax:	0511 532-7276

fuchs.heiko@mh-hannover.de

Carl-Neuberg-Straße 1

30625 Hannover

Telefon: 0511 532-0

www.mh-hannover.de

Dear reviewers,

23. Nov. 2023

First, we thank the reviewers for taking the time to read and review our manuscript. We have improved the manuscript based on their comments and suggestions and would like to address their points in detail below.

**Responses to the Reviewer #1 comments.**

*In this highly interesting manuscript, the authors describe a novel method for in vitro monitoring of AAV transduction, by using GFP transduction and Hoechst 33342 staining. The authors present a very useful method with the possibility of serial measurements. All results are sound and the conclusions drawn are convincing. The linguistic style is good, which makes the manuscript easy to read and understand. The work presented will be of high interest to your readers. Therefore I strongly recommend publication, without further changes to the manuscript necessary.*

**Answer:**

We thank the reviewer for the positive comment.

**Responses to the Reviewer #2 comments.**

*This study employed a simple protocol combining Hst33342 and a transgene reporter (GFP) to track AAV transduction efficiency during 2D cell culture. This involved quantifying imaged cells in both primary human RPE and RPE cell lines. This procedure equipped by microscope and image analysis with live cell and GFP positive cell counting would be straightforward. While the gathered data is intriguing, there is room for improvement in the study's design to better elucidate its novelty and feasibility.*

*Reviewer suggested a major revision with following comments and questions.*

*Major Revisions:*

*1. Clarify whether hst33342 is added one-time at seeding or recharged during long-term culture with medium replacement.*

**Answer:**

1. 28 nM Hoechst 33342 was added every five days. We clarified this in the method (Line 107): **"** **The medium was changed every 3 days. 28 nM Hoechst was added every five days."**
2. The dosage of Hoechst was revised due to a calculation mistake. 4 ng/10^4^ cells, instead of 6 ng/10^4^ cells, Hoechst corresponds to 28 nM (Line 101): **"Four ng/104 cells of Hoechst, which corresponds to a concentration of 28 nM that can be used in live cells for long-term staining, according to our previous study [19], were added to stain the nuclei."**

*2. line 308: In addition to utilize hst33342 as a live cell marker, with transgene GFP, which serves as a visible marker for monitoring AAV transduction, author need to collect more data to show the benefit or superiority of Hst33342 application. Additionally, using Gen5 Image Prime 3.05 (Santa Clara, CA, U.S.) software for image analysis may be less valuable if it still in market, compared to other free and open-source software like Image J. Author need to state the equipment and software can be adopted to universal microscope imager.*

**Answer:**

1. We added two figures to compare Hoechst cell counting and normal phase contrast counting. We elaborated on the superiority of Hoechst cell counting in the discussion (Line 371): **"Usually, cell bodies are gated for automated counting when nuclei are not stained. However, the meniscus effect reduces the contrast between cells and the background at the periphery in PC images and is more pronounced in smaller diameter wells such as 48 and 96-well plates (Fig 5A). Due to the overexposure at the outer edges of the image, cells are hardly recognizable. They cannot be optically separated from the background, making an additional background processing step necessary, which requires additional computing time and storage capacity. Even after background processing, it is difficult to detect the cell bodies depending on the cell type (Fig 5B). For example, cells with an irregular body shape, such as fibroblast-like cells, are much more challenging to be correctly gated. Parameters used for image preprocessing and gating also vary between users, leading to user-dependent results. In Fig 5, we compared automated and Hoechst counts on hSF cultivated in a 96-well format. With automated count, cells are challenging to recognize separately, and the object gating of cell bodies is unreliable, showing a significant discrepancy with the Hoechst count of the nuclei. 504 events were identified with automated cell counting, whereas only 159 nuclei were counted in the Hoechst channel. The sub-mask of the automated cell count revealed 182 GFP-positive hSFs in the GFP channel, while the Hoechst count identified only 81 GFP-positive hSFs (Fig 5B+C). In the lower left picture of panel B, many primary masks generated from automated cell counting included no nuclei. Since the automated cell count for hSF is imprecise, the percentage of GFP-positive cells and the calculated transduction time 50 (TT50) are incorrect compared to the Hoechst-gated nuclei. (Fig 5D+E). With the assistance of Hoechst, fluorescent nuclei, which are usually more regular in size and spatially separated from each other, appear more prominent in the background and can be gated more efficiently."**
2. This method can be adopted with different microscopes and software apart from Gen5, which is only an example in this study (Line 398): **"Our approach can be used either with an inverted fluorescence microscope or a live-cell imager combined with microplate analysis software."** Additionally, we tested some of the images using ImageJ and compared the results with our method. Line 249: **"In addition, the images were analyzed with ImageJ software (National Institutes of Health, Bethesda, MD,** [**https://ij.imjoy.io/**](https://ij.imjoy.io/)**) to validate the accuracy of our method. The results showed no significant difference in transduction efficiency with our proposed method (S1 Fig)."** Unfortunately, it is unrealistic for us to test it with all software in market. However, the principle and workflow of gating nuclei, determining GFP signal intensity, and calculating GFP-positive cells should work with most analysis software. This study aims to offer a new idea for monitoring transduction efficiency in real time in live cells using Hoechst.

*3. While the procedure with hst33342 to monitor AAV transduction has been developed using human primary cell RPEs, it seems suitable only for 2D adhered cell culture. Please discuss the applicability of this method to other primary cells in suspension or 3D conditions.*

**Answer:**

Unfortunately, this method is unsuitable for cells in suspension or 3D conditions. The limitation was clarified in the discussion already (Line 427): **"However, this method is not suitable for cells that are not at the same focal level, for example, suspension cells and organoids."**

*4. line 312: For high throughput, consider using 96 or 384 well plates instead of the 48 well plate format. Additionally, explore the possibility of automated or programmable analysis for real-time monitoring of AAV transduction. Reducing the number of cells in 96/384 plate could also streamline data analysis.*

**Answer:**

1. We added an experiment of transducing human scleral fibroblasts with AAV2 in a 96-well plate for 5 days to verify that this method can be applied in other plate formats (Line 315): **"Finally, to emphasize the advantages of our method, we chose hSFs, which vary considerably in size and shape. We performed the transduction with AAV2-CMV-GFP at MOIs of zero, 2.5 x104, 5 x104, and 10 x104 in 96-well format, often used for high-throughput analyses and monitored continuously for 120 hours (Fig 5)."**
2. Multi-well plates such as 48 and 96-well for high-throughput analysis are used. However, due to the meniscus effect, cell bodies become less prominent and overexposed at the periphery in smaller diameter wells (Fig 5A). This effect makes it difficult to use automated phase contrast to count cell numbers even after a background subtraction step. Also, automated cell gating and counting are unreliable for irregular cell shapes. In the lower-left picture in Fig 5B, many primary masks do not include nuclei inside, proving to be unreliable. The discussion (Line 371) and the previous comment explained the inferiority of automated count.

*5. To achieve long-term monitoring of AAV transduction, consider extending the duration beyond 4 days post-AAV infection. The authors might also contemplate transitioning this method from cell culture to ex vivo or in vivo models for more meaningful insights.*

**Answer:**

1. Although we only presented images at certain time points, the transduction experiment in hRPE lasted for 14 days (Line 266): **"First, we compared different serotypes in the same cell type by transducing AAV2-CMV-GFP, AAV6-CMV-GFP, and AAVDJ-CMV-GFP at the same MOI in hRPE cells and monitored them for 14 days."** In Fig 3, the tendency of transduction efficiencies over two weeks was shown in a bar chart.
2. One of the limitations is that this method cannot be adopted in organoids, either ex vivo or in vivo models, which was clarified in the discussion (Line 427): **"However, this method is not suitable for cells that are not at the same focal level, for example, suspension cells and organoids."**

*6. For cell types with multiple nuclei, such as primary human hepatocytes, integrating intelligent software programs to outline cell borders could enhance the significance of this work.*

**Answer:**

1. Due to the meniscus effect and depending on the cell type, it is challenging to outline the cell border in multi-well plates even after background processing. There is a great deal of subjectivity and error in this process. However, Hoechst can stain the nuclei and make cell counting more manageable and objective because fluorescent nuclei are prominent in the dark background and separate from each other. The disadvantages of automated count and the superiority of Hoechst count were elaborated in the discussion (Line 371) and the previous comment.
2. This is a limitation of our method that it cannot be adopted in multi-nuclei cells and we added this in the discussion (Line 428): **"It is trivial, but it should be mentioned that cell counting with Hoechst only works with mononuclear cells."**

*Minor Revisions:*

*1. Fig1A: please use another icon to represent AAV virions.*

**Answer:**

The icon of AAV was changed in Fig 1.

*2. For each figure, please indicate each color.*

**Answer:**

In each figure or figure legend, the meaningful color was already clearly indicated, e.g.,**"Hoechst-stained nuclei labeled in blue indicated GFP-negative, and the ones marked in red indicated GFP-positive cells."** Other colors are used for aesthetic purposes only, which in the opinion of the authors, are not necessary.

*3. Line 71: Could you please provide the full name of "hRPE" at its first mention in the manuscript? The same applies to "ARPE" later in the text.*

**Answer:**

The definitions of the terms were added at the first mention (Line 76 and 98): **"Human retinal pigment epithelium (hRPE) cells and human scleral fibroblasts (hSF) were isolated from patients undergoing enucleation."** and **"1 x104 hRPE cells and the spontaneously arising RPE cell line, ARPE-19 cells, were seeded "**.

*4. It is recommended to enhance the quality of almost all figures to ensure they are easily recognizable and comprehensible.*

**Answer:**

Due to technical reasons, the sharpness of all figures declined after submitting them to the online system. Nevertheless, a high resolution picture can be downloaded by clicking the link above the Figures in the PDF document.

*5. line 149: considering the GFP intensity not saturated, the exposure time also need to be optimized for each batch of AAV transduction.*

**Answer:**

Although the GFP signal is not saturated at the beginning, the exposure time can be set relatively longer to ensure a sensitive detection of GFP signal. The images' backgrounds can be subtracted afterward in analysis. Also, the histogram of each channel can be adjusted as long as the same capture parameters are applied. Nevertheless, a pretest can be considered to figure out an optimized acquisition time.

*6. The reviewer is interested in whether the signal from hst33342 or the GFP signal in the image-based analysis can be correlated with the readings from the plate reader, which is less time-consuming.*

**Answer:**

Plate readers equipped with an integrated inverted microscope, which can record individual cells and nuclei, are suitable for our method. Plate readers that do not have this feature can only determine the average intensity for Hoechst and GFP but cannot provide any information about the transduction efficiency.

**Responses to the Reviewer #3 comments**

*In this manuscript, the authors propose a method for real-time detection of AAV transfection efficiency by pre-treating cells with Hoechst 33342, a DNA fluorescent dye, before transfection. The treated cells were then analyzed using specialized instruments for real-time monitoring. The authors experimentally validated the feasibility of this method and compared it with traditional methods.*

*Main Suggestions:*

*The introduction section lacks sufficient information about Hoechst 33342. Please provide a detailed explanation of Hoechst 33342 in the Introduction to offer readers a comprehensive understanding of its role in the experiment.*

**Answer:**

A more detailed introduction of Hoechst 33342 was added (Line 62): **"For accurate cell counting, Hoechst 33342 (Hoechst) was added to the cells before the transduction. Hoechst is a DNA-binding fluorochrome that can stain the fluorescent blue nuclei in both live and dead cells. It is widely used in endpoint analysis at a high concentration due to its binding with the DNA groove [18]. However, according to our previous work, a concentration between 7 to 28 nM is neither cytotoxic nor inhibits cellular proliferation and provides a sufficient staining of cell nuclei. In addition, the acquisition parameters were optimized to exclude possible phototoxic effects due to the excitation light [19]."**

*Clarify how the minimal dosage of Hoechst 33342, used as a nuclear stain, was determined in this experiment. Provide the rationale or methodology behind selecting this specific dosage.*

**Answer:**

1. The reason of the dosage of Hoechst was added in the discussion (Line 366): **"In previous work, we have shown that by optimizing the acquisition parameters, a low Hoechst concentration in the medium is sufficient to visualize the nuclei over five days without significantly affecting cell activities such as cell proliferation or signaling cascades. Although the appropriate Hoechst concentration varies from cell type to cell type, a concentration of 7 to 28 nM is recommended based on our previous study [19]."** To show a stronger contrast of the nuclei, 28 nM Hoechst was applied.
2. The dosage of Hoechst was revised due to a calculation mistake. 4 ng/10^4^ cells, instead of 6 ng/10^4^ cells, Hoechst corresponds to 28 nM (Line 101): **"Four ng/104 cells of Hoechst, which corresponds to a concentration of 28 nM that can be used in live cells for long-term staining, according to our previous study [19], were added to stain the nuclei."**

*The manuscript only mentions the use of RPE cells. Explain the reason for choosing this specific cell line. However, the authors did not investigate the effectiveness of this method on other cell types, making the conclusion overly optimistic. It is recommended to justify the choice of ocular cells and provide a background on current AAV research and treatments related to ocular diseases. Relevant studies such as PMID: 37273779 and PMID: 35408825 can be cited to support the rationale.*

**Answer:**

1. AAV transduction is a widely used approach in various disciplines focusing on all kinds of cells, including ocular cells. The choice of ocular cells is based on the nature of our laboratory. We used both human RPE cells and the cell line ARPE-19 to indicate that this method can be applied in both primary cells and cell lines.
2. We added an experiment of transducing AAV2 in human scleral fibroblasts to show that this method can be used in other cell type (Line 315): **"Finally, to emphasize the advantages of our method, we chose hSFs, which vary considerably in size and shape. We performed the transduction with AAV2-CMV-GFP at MOIs of zero, 2.5 x104, 5 x104, and 10 x104 in 96-well format, often used for high-throughput analyses and monitored continuously for 120 hours (Fig 5)."** However, it is unrealistic to validate this method in all cell types. The purpose of our study is to offer a new idea to monitoring transduction efficiency in real-time in live cells with the assistance of Hoechst.

*The manuscript lacks a seamless integration between the methods and images. Many instances merely state that the method can be applied without immediate validation from experimental data. To enhance the manuscript's credibility, include experimental data comparisons between this method and traditional approaches. This will allow readers to visualize the advantages of this method.*

**Answer:**

1. To validate the accuracy, we used ImageJ to analyze the images of hRPE cells transduced with AAV2 after 24 h. The representative pictures were shown in S1 Fig. A t-test indicated that the transduction efficiencies calculated with two methods had no significant difference. Line 249: **"In addition, the images were analyzed with ImageJ software (National Institutes of Health, Bethesda, MD,** [**https://ij.imjoy.io/**](https://ij.imjoy.io/)**) to validate the accuracy of our method. The results showed no significant difference in transduction efficiency with our proposed method (S1 Fig)."** However, there is no gold standard so far. ImageJ is only a commonly used method.
2. In addition, nuclei counting after fluorescent staining is relatively reliable today. In Fig 5 and S2 Fig, we compared traditional automated count and Hoechst count. In phase contrast images, meniscus effect causes it difficult to recognize cell bodies and requires additional background subtraction. However, the processing is user-dependent and cell type-dependent. The outline of cell borders is not trustworthy even after background processing. The drawback of traditional automated counting and the reliability of Hoechst counting was elaborated in the discussion (Line 371): **"Usually, cell bodies are gated for automated counting when nuclei are not stained. However, the meniscus effect reduces the contrast between cells and the background at the periphery in PC images and is more pronounced in smaller diameter wells such as 48 and 96-well plates (Fig 5A). Due to the overexposure at the outer edges of the image, cells are hardly recognizable. They cannot be optically separated from the background, making an additional background processing step necessary, which requires additional computing time and storage capacity. Even after background processing, it is difficult to detect the cell bodies depending on the cell type (Fig 5B). For example, cells with an irregular body shape, such as fibroblast-like cells, are much more challenging to be correctly gated. Parameters used for image preprocessing and gating also vary between users, leading to user-dependent results. In Fig 5, we compared automated and Hoechst counts on hSF cultivated in a 96-well format. With automated count, cells are challenging to recognize separately, and the object gating of cell bodies is unreliable, showing a significant discrepancy with the Hoechst count of the nuclei. 504 events were identified with automated cell counting, whereas only 159 nuclei were counted in the Hoechst channel. The sub-mask of the automated cell count revealed 182 GFP-positive hSFs in the GFP channel, while the Hoechst count identified only 81 GFP-positive hSFs (Fig 5B+C). In the lower left picture of panel B, many primary masks generated from automated cell counting included no nuclei. Since the automated cell count for hSF is imprecise, the percentage of GFP-positive cells and the calculated transduction time 50 (TT50) are incorrect compared to the Hoechst-gated nuclei. (Fig 5D+E). With the assistance of Hoechst, fluorescent nuclei, which are usually more regular in size and spatially separated from each other, appear more prominent in the background and can be gated more efficiently."**

*Provide a detailed discussion on how the accuracy of this method was validated. Readers require comprehensive insights into the validation process, including methodologies and results, to fully comprehend the reliability of the proposed technique.*

**Answer:**

We added an analysis of the images of hRPE cells transduced with AAV2 after 24 h using ImageJ (Line 249): **"In addition, the images were analyzed with ImageJ software (National Institutes of Health, Bethesda, MD,** [**https://ij.imjoy.io/**](https://ij.imjoy.io/)**) to validate the accuracy of our method. The results showed no significant difference in transduction efficiency with our proposed method (S1 Fig)."** Nevertheless, there is no gold standard to validate the accuracy of our method today. The superiority of nuclei counting with Hoechst was elaborated in the discussion (Line 371) and in the previous comment.
