# Supplementary material for: Long-term in vitro monitoring of AAV-transduction efficiencies in real-time with Hoechst 33342
Source: PLoS One. 2024 Mar 1;19(3):e0298173. doi: 10.1371/journal.pone.0298173 (PMC10906819; doi:10.1371/journal.pone.0298173)
Supplement: Attachment — Submitted filename: Response to Reviewers.docx [file pone.0298173.s006.docx]

**Forschungslabor Experimentelle Augenheilkunde**

**Dr. rer. nat Heiko Fuchs, Laborleiter**

OE6120

Heiko Fuchs

Telefon:	0511 532-7246

Fax:	0511 532-7276

fuchs.heiko@mh-hannover.de

Carl-Neuberg-Straße 1

30625 Hannover

Telefon: 0511 532-0

www.mh-hannover.de

Dear reviewers,

04. Jan. 2024

We appreciate your comments and made necessary adjustments based on the suggestions. We would like to address the points in detail below.

**Responses to Reviewer #2 comments.**

*There are notable concerns regarding the accurate differentiation between dying or dead cells and debris from live cells in images without manual evaluation. However, the addition of a single drop of propidium iodide (PI) enables the exclusion of debris or dead cells in flow cytometry. At present, there seems to be no compelling advantage over flow cytometry. Even with the capacity of a 96T plate reader, detecting fewer cells from various time points under identical settings is feasible. Image capture and analysis might require a longer operation and analysis compared to flow cytometry.*

**Answer:**

1. PI is a dead-cell dye that is not only used in flow cytometry. PI can be also dopted in our method to differentiate dead cells or debris, as demonstrated in our prior publication. Hoechst-stained objects are well suited to be used as primary masks in a red channel, allowing the identification of PI-positive cells. The detection and calculation is the same as GFP-positive cells. The detailed method is added in the discussion (Line 409): **"Considering that Hoechst also stains dead cells, other dyes that specifically stain dead cells or debris, such as propidium iodide (PI), can be applied simultaneously, as demonstrated in our prior publication [19]. PI-positive cells are detected with a red fluorescence filter and sub-masked within the primary Hoechst-stained objects. The number of viable cells is calculated by subtracting the number of PI-positive nuclei from Hoechst-positive nuclei. The transduction efficiency can be measured using the number of viable cells instead of total Hoechst-positive nuclei for a more accurate evaluation."**
2. Manual evaluation is impractical in cases of abundant cell numbers, irregular cell shapes, and colonies. In Fig 5, the phase contrast image and its processed images indicate that manual counting is unsuitable. The disadvantages have already been clarified in the discussion (Line 382).
3. After repeating the method in a 96-well plate as requested in the first revision, we are sorry that you don’t see the advantages over flow cytometry. The disadvantages of flow cytometry were addressed in the manuscript, namely that it is an endpoint analysis that is time-consuming and materials-consuming (Line 363). In contrast, these are precisely the advantages of our method. Although they were clarified in the discussion already, we would like to emphasize again:
1) Our method is not limited to an endpoint analysis. It allows transduction efficiencies to be repeatedly measured over several days at any desired time point. (Line 439)
2) Using a 48- or 96-well plate allows evaluating and monitoring multiple transduction parameters in one run, such as viral serotypes, MOIs, cell types and cell numbers. (Line 434)
3) For example, for a plate tested at 30-minute intervals for 72 hours, 145-time points per well can be analyzed in our method. However, using flow cytometry, 145 experiments are needed to determine these 145 time points. At your request, we have repeated our analysis in 96-well format. How many wells would be needed to monitor 96 conditions in 30-minute intervals for 72 h by flow cytometry? Exactly 96 x 145 wells.
4) Even if labeling dead cells (e.g. with PI) makes sense in some cases, transduction with AAV can impair the viability of cells. Nevertheless, a reduced cell viability does not necessarily lead to cell death but rather to a reduced proliferation rate, as shown in Fig 4, where we tested different MOIs. Can you also determine the proliferation rates in real-time with flow cytometry, as we have shown with our method? Therefore, we included in Line 417:**“Nevertheless, cytotoxicity does not necessarily lead to the death of cells but can also manifest itself through reduced viability, like a reduced proliferation rate. One advantage of our method is that not only the transduction rates but also the proliferation rates can be determined simultaneously. For example, ARPE-19 transduced with AAV-DJ-CMV-GFP at an MOI of only 5x104 vg/cell show a significant inhibition of proliferation and, thus, impairment of their cell viability (Fig. 4 C).”**
5) As the manuscript outlines, utilizing multiple images per well allows for a more detailed analysis, contributing to a comprehensive understanding of the experimental results. However, it is worth noting that there are no significant differences in transduction efficiencies, whether analyzing 3000 or “only” 1000 cells.
6) Compared to flow cytometry, our method requires less time and less materials, including fewer cells, fewer cell media, less viral particles, and therefore is more resource-efficient and user-friendly.
4. It is fast to perform one run because the analysis is used as an automated program protocol. With a more sophisticated machine, such as a live-cell imager, image capture is also automated, and it takes around 30 minutes to test a 48-well plate. In addition, our method eliminates the need to harvest cells and prepare samples.

*1. Line 101: It is imperative to provide the product information of Hst 33342, including details about the solvent, storage conditions, and shelf life for the execution of this work.*

**Answer:**

The corresponding information of Hoechst 33342 was added in Line 98: **“Hoechst (Sigma-Aldrich B2261-25MG) was dissolved in sterile double distilled water to a concentration of 1 mg/ml and sterile-filtered using a 33 mm diameter PES syringe filter with a pore size of 0.22 µm. The solution was aliquoted and stored at -20°C. In experiments, the solution was diluted in sterile double distilled water or medium and added directly into the cell medium.”**

*2. Line 103: We typically utilize AAV-D/J or AAV-DJ instead of AAVDJ.*

**Answer:**

The names of AAV in the manuscript and figures were changed. For example, from “AAVDJ” to “AAV-DJ”.

*3. Line 104: Please specify the AAV type as ss or sc?*

**Answer:**

In our study, we used scAAV to transduce cells and have included this information in the method section. However, we provide a better approach to measure transduction efficiencies, and the choice of viral vectors depends on the researcher.

*4. Line 106: Our standard practice involves using the Multiplicity of Infection (MOI) as vg/cell for AAV application in vitro.*

**Answer:**

The unit was added in Line 114: **“For hRPE cells, AAV-2-CMV-GFP, AAV-6-CMV-GFP, and AAV-DJ-CMV-GFP particles purchased from Charles River (Wilmington, MA, U.S.) were suspended in the same medium and added into cells at a multiplicity of infection (MOI) of 5 x104 GC/cell (the following units are identical).”** The following MOI units were omitted since the unit of MOI is normally omitted.

*5. Line 108: Was the medium completely replaced at 100%?*

**Answer:**

Yes, the medium was replaced entirely. This statement was added as required. Line 115: **“The medium was completely changed every 3 days.”**

**Responses to Reviewer #3 comments**

*In general, the author has addressed the feedback effectively by providing additional information, justifications, and experimental data where necessary. The manuscript appears to have been improved based on the feedback received.*

*Other concerns are as follows:
Adding more literature will make the manuscript more comprehensive. The author did not cite the correct literature. (refer to previous review comments 3)*

**Answer:**

As previously explained, our study aims to offer a new idea for monitoring transduction efficiency in real-time in live cells using Hoechst. Therefore, the choice of cell types and viral vectors depends on the researcher, and the method is not limited to ocular cells and AAV. It is pointless and weird to emphasize current AAV research in ophthalmology because it was not our focus. Nevertheless, as you requested, we have included your previously published article (PMID: 37273779) and the other article you mentioned (PMID: 35408825) in the reference list, stating in Line 370: **“Since AAV vectors are one of the established transduction approaches applied in diseases related to the eye, liver, muscle, and nervous system and have numerous serotypes with different affinities to cells, we chose AAV vectors to transduce cells to exhibit the advantages of our method [1,2,23–25].”**

We hope that our revised version will be published in PlosOne and remain with kind regards.

Heiko Fuchs

Corresponding Author
